# The presence of pathogens and heavy metals in urban peregrine falcons (*Falco peregrinus*)

**DOI:** 10.14202/vetworld.2021.1741-1751

**Published:** 2021-07-03

**Authors:** Ewelina Pyzik, Marta Dec, Dagmara Stępień–Pyśniak, Agnieszka Marek, Jose Louis Valverde Piedra, Agnieszka Chałabis-Mazurek, Klaudiusz Szczepaniak, Renata Urban-Chmiel

**Affiliations:** 1 Department of Veterinary Prevention and Avian Diseases, Faculty of Veterinary Medicine, University of Life Sciences, Akademicka 12, 20-033 Lublin, Poland; 2 Department of Preclinical Veterinary Sciences, Faculty of Veterinary Medicine, University of Life Sciences in Lublin, Lublin, Poland; 3 Department of Veterinary Parasitology and Fish Diseases, Faculty of Veterinary Medicine, University of Life Sciences, Akademicka 12, 20-033 Lublin, Poland

**Keywords:** bacteria, falcons, health status, toxic elements, welfare

## Abstract

**Background and Aim:**

Wild birds raised in urban environments may be exposed to many negative factors, including biological and chemical toxic elements. The aim of the study was to assess the occurrence of bacteria and parasites in wild birds, based on the example of the peregrine falcon (*Falco peregrinus*) as a potential indicator of bacterial drug resistance genes. Toxicological contamination was also analyzed to determine the impact of urbanized areas on this predatory species, in terms of its health, welfare, and survival in urban environments.

**Materials and Methods:**

The samples consisted of down feathers and fresh feces obtained from seven falcon chicks (during obligatory veterinary examination) reared in two nests located in the Lublin region (Lublin and Puławy). Bacteria and parasites were isolated directly from feces by classical microbiological methods, polymerase chain reaction, and matrix-assisted laser desorption/ionization time-of-flight mass spectrometry (MS). The down feathers and feces of birds were used for toxicological testing by plasma inductively coupled plasma MS to assess the concentrations of selected heavy metals (cadmium [Cd], lead [Pb], arsenic [As], zinc [Zn], and copper [Cu]).

**Results:**

The study revealed the presence of a diverse microbiome in the falcon chicks, among which *Escherichia coli*, *Enterococcus* spp., and *Staphylococcus* spp. bacteria and parasites of the genus *Caryospora* were dominant. The presence of drug resistance genes was also confirmed among the pathogens. The toxicological analysis found high concentrations of toxic heavy metals, including Cd, Pb, As, and Zn, in the downy feathers and feces of peregrine chicks.

**Conclusion:**

Predatory free-living birds living in urban environments not only can be infected with various pathogens but may also show contamination with heavy metals, which could influence their natural resistance, condition, and welfare.

## Introduction

Wild birds, due to their wide geographical range and close contact with humans, may be a potential reservoir of microorganisms with antibiotic resistance genes [1]. Their presence within human settlements and contact with chemical agents, antibiotics, sulfonamides, and other toxic substances may have a significant impact on the composition of their microbiota as well as their exposure to heavy metals or other toxins. Wild migratory birds in temperate climates have been shown to have the most diverse bacterial microbiota, in which the most commonly isolated bacteria include strains of *Escherichia coli*, *Salmonella*, *Campylobacter*, *Pasteurella multocida*, and *Borrelia burgdorferi* [1,2]. It has been postulated that wild birds are reservoirs and potential carriers of antibiotic resistance genes. Numerous studies have confirmed the role of free-living birds in the prevalence of multidrug-resistant bacteria, which could be a significant pathway in transmission to other animals and humans [3,4]. Monitoring of the spatial and temporal distribution of resistant bacteria in wild birds can be an important diagnostic indicator in the control of drug-resistance vectors and thus help to reduce the growing global problem of antibiotic resistance.

The peregrine falcon (*Falco peregrinus*) is one of the most widespread raptors in the world and is found on all continents, including Antarctica. In Europe, it currently inhabits primarily the western and southern parts of the continent, including Great Britain, France, and Spain, as well as northern Scandinavia and Russia [5,6]. Due to food conditions, fairly close contact with livestock or companion animals, and indirect contact with humans, the peregrine falcon may be exposed to human pathogenic microorganisms, including *Campylobacter* spp., *Salmonella*, *E. coli* or *Mycoplasma* [7-9]. Due to close contact with rapidly developing urban environments, peregrine falcons can also be exposed to toxic elements such as cadmium (Cd), copper (Cu), and lead (Pb), which can affect their health status, welfare, and immunity, with increased susceptibility to infection. Concentrations of toxic heavy metals in the organs of free-living birds may Pb to their death in regions contaminated with these elements [10,11]. Heavy metals enter the environment and undergo biomagnification in high trophic level organisms, in which they can reach toxic concentrations [12]. As environmental pollution increases, so does exposure of wildlife to xenobiotics (chemical substances not naturally produced by organisms), including highly toxic metals. One of the important chemical environmental pollutants, toxic even in low concentrations, is Cd, which due to its ease of absorption and capacity for bioaccumulation in living organisms and biomagnification in the food chain poses a major threat to animals and humans health [10]. The mechanism of Cd toxicity is induction of oxidative stress and lipid peroxidation of cell membranes, which Pbs to changes in the metabolism and structure of cells and irreversible damage to them [11]. In the case of mercury (Hg), which has the highest accumulation factor, toxicity poses a significant risk not only to humans and animals but also to ecosystems as well [12]. Pb is another widespread element in nature that does not undergo biodegradation or decomposition in the natural environment and accumulates in tissues [13].

The aim of the study was to assess the occurrence of potential pathogens and antibiotic resistance genes in bacteria isolated from peregrine falcons (*F. peregrinus*) that nest in the vicinity of humans. Given the potential hazards arising from increasing pollution of the environment, the concentrations of heavy metals known to be present in urban environments were determined in the feathers and feces of the birds.

## Materials and Methods

### Ethical approval

Ethical approval is not necessary for such type of study; however, samples were collected as per standard sample collection procedure without any harm to the birds.

### Study period and location

The study was conducted in March 2019. The samples were collected from Lublin and Puławy of Poland. The samples were processed at Department of Veterinary Prevention and Avian Diseases, Faculty of Veterinary Medicine, University of Life Sciences in Lublin.

### Sample collections

**Figure-1:**
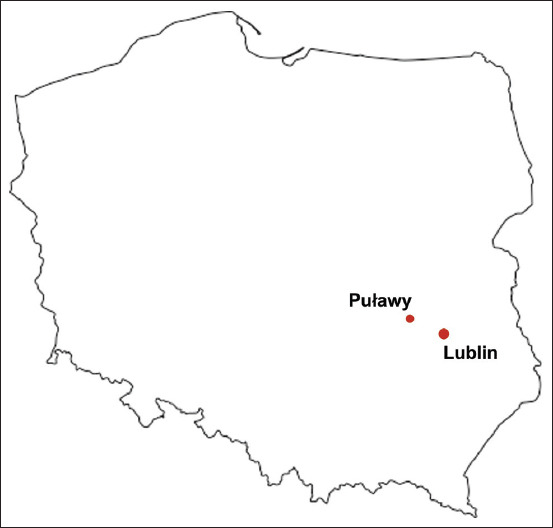
The map with the location of the peregrine falcons’ nests.

The study material consisted of fresh feces and down feathers collected from seven young falcons from two nests located in urban areas of the Lublin region (Lublin and Puławy) (Figure-1). The material was collected from 3-week-old chicks during obligatory veterinary examination and ringing. The average weight of the chicks from the nest in Lublin was 831 g (male – 645 g and female 1 – 955g; female 2 – 893 g), while the average weight of the chicks from Puławy was 833 g (male – 700 g; females 1 and 2 – 900 g; female 3 – 899 g). The material was delivered to the laboratory within 2 h in conditions compatible with transport standards (in sealed bags in a sample refrigerator). Parasitological diagnostics (phenotypic analysis) and bacteriological analysis (phenotypic analysis, polymerase chain reaction [PCR], and matrix-assisted laser desorption/ionization time-of-flight mass spectrometry (MALDI TOF MS) of the feces were carried out, as well as toxicological analysis by inductively coupled plasma MS (ICP-MS).

### Isolation and identification of bacteria

The bacteria were identified by MALDI-TOF MS using a standard extraction method [14,15], with an UltrafleXtreme MALDI TOF mass spectrometer (Bruker, Germany). The mass spectra obtained from each isolate were processed with MALDI Biotyper 3.0 software (Bruker, Germany), and the results were shown as the top ten identification matches together with confidence scores (similarity of species) ranging from 0.000 to 3.000, calculated by comparing the reference main spectra. According to the manufacturer’s criteria, a log score <1.700 does not allow for reliable identification; a log score between 1.700 and 1.999 allows identification to the genus level; a log score between 2.000 and 2.299 means highly probable identification at the genus level and probable identification at the species level; and a log score >2.300 (2.300-3.000) indicates highly probable identification at the species level.

The *E. coli* strains were isolated on MacConkey agar, and pure cultures were stored on brain heart infusion (BHI) broth with ~20% glycerol. To determine the phylogenetic groups of the isolates, five sets of primers for the genes *yja*, *TspE4.C2*, *chuA*, *svg*, and *uidA* were used in multiplex PCR [16]. Amplicons were separated by electrophoresis in 3% (wt/vol) high resolution agarose (Blirt, Poland). The phylogenetic groups (A, B1, B2, and D) were determined based on the PCR gel pattern.

Antibiotic susceptibility was determined by agar disk diffusion according to the guidelines of the Clinical and Laboratory Standards Institute [17] on Mueller-Hinton agar plates (Oxoid, UK), followed by application of antibiotic discs: Ampicillin (AM, 10 μg), cefotaxime (CTX, 30 μg), imipenem (IPM, 10 μg), gentamicin (CN, 10 μg), kanamycin (K, 30 μg), amikacin (AK, 30 μg) chloramphenicol (C, 30 μg), tetracycline (TE, 30 μg), trimethoprim (W, 5 μg), and ciprofloxacin (CIP, 5 μg). Inhibition zone diameters were measured after incubation at 35°C for 16-20 h, and the results were interpreted according to the breakpoint recommended by the CLSI [17]. Antibiotic resistance genes, including *tet* genes (*tet*(A), *tet*(B), and *tet*(C)), were detected in isolates with phenotypic tetracycline resistance by multiplex PCR [18]. PCR reactions were used to detect the presence of 24 genes associated with virulence in *E*. *coli* strains. The antibiotic resistance genes were determined on the basis of phenotypic tetracycline resistance (Table-1) [19-22]**.** PCR products were separated by electrophoresis (100 V) on 2% agarose gels, visualized by staining with SimplySafe (Eurx, Poland), and documented with the GelDoc system, using Quantity One software (Bio-Rad, Germany).

**Table-1:** Virulence genes determined in *Escherichia coli* isolates based on literature data.

PCR method	Detected genes	References
Multiplex 1	*stx1, stx2, hylA, eaeA, saa*	Paton and Paton [20]
Multiplex 2	*ecsV, ent, bfpB, invE, astA, aggR, pic, ipaH, elt, estIa, estIb*	Turchi *et al*. [19]
Multiplex 3	*ompT, iutA*	van der Westhuizen and Bragg [21]
Multiplex 4	*tsh, pap-C, iss, irp-2*	Ewers *et al*. [22]
Uniplex 1	*cva/cvi*	Ewers *et al*. [22]
Uniplex 2	*iucD*	Ewers *et al*. [22]

PCR=Polymerase chain reaction

Directly after collection, the bacteria were streaked onto Bile Esculin Azide Lab-Agar (Biocorp, Poland), a selective-differential medium for *Enterococcus*, supplemented with 5% horse blood (ProAnimali, Poland), and incubated for 24-48 h at 37°C. Colonies with typical enterococcal morphology were selected to identify specific *Enterococcus* species, and following incubation in BHI broth (Oxoid, UK), the isolates were stored at 80°C for further analysis. The enterococcal isolates were identified using MALDI-TOF MS [23]. The identification was confirmed by sequencing of the *rpoA* gene [24].

The susceptibility of the *Enterococcus* isolates was tested for 17 antibiotics: AM, cefotaxime (CTX, 30 μg), IPM, CN, AK, chloramphenicol (C, 30 μg), TE, sulfamethoxazole/trimethoprim (SXT, 25 μg), vancomycin (VA, 30 μg), penicillin (P, 10 μg), streptomycin (S, 25μg), erythromycin (E, 15 μg), lincomycin (LS, 109 μg), tigecycline (TGC, 15 μg), teicoplanin (TEC, 30 μg), and linezolid (LZD, 30 μg). Inhibition diameter zones were measured after incubation at 35°C for 16-20 h, and the results were interpreted according to CLSI [17]. *Enterococcus faecalis* ATCC 29212 was used as quality control.

Genomic DNA was extracted using the commercial GeneMATRIX Bacterial and Yeast Genomic DNA Purification Kit (Eurx, Gdansk, Poland). Antibiotic-resistant isolates were tested by PCR for detection of the following resistance genes: *vanA*, *vanB*, *vanC1*, *vanC2*/*C3*, *vanD*, *vanE*, and *vanG* (glycopeptide-resistant isolates), *blaZ* and *pbp5* (penicillin- and ampicillin-resistant), *aph(30)-*IIIa (kanamycin-resistant), *ant(6)-*Ia (streptomycin-resistant), *tet*(M), *tet*(L), *tet*(K), and *tet*(O) (tetracycline-resistant), and *erm*(A), *erm*(B), *msr*(A/B), and *msr*(C) (macrolide-resistant) [25].

The bacterial strains were isolated on mannitol agar (Chapman medium) and Columbia agar with 5% sheep blood at 37°C for 24 h. The resulting cultures were incubated in TSB broth (BTL and PL) at 37°C for 24 h to obtain optimal growth of pure strains in a liquid medium. Phenotypic identification of the bacterial isolates was carried out by Gram staining. Genetic identification was carried out by PCR and MALDI-TOF MS [26]. The drug-susceptibility of bacteria was determined by the agar disk diffusion method CLSI [17], followed by application of antibiotic discs (Oxoid, UK): Amoxicillin (AMC 30 μg), amoxicillin+clavulanic acid (AMC 30 μg) cefoxitin (FOX 30 μg), clindamycin (DA 2 μg), enrofloxacin (ENR 5 μg), E, CN, LZD, neomycin (N 30 μg), VA, CIP, C, STX, P, polymyxin (0.5-256 μg/mL), TEC, TE, and tobramycin (TOB 10 μg). *Staphylococcus aureus* ATCC 25923 and *S. aureus* ATCC 29213 were used for quality control.

Identification of selected antibiotic resistance genes, *tet*(K), *bla*Z, *walKR*, *vraSR*, and *rpoB*, was carried out for multidrug-resistant *Staphylococcus* spp. by multi-PCR [27,28]. The strains were tested for genes encoding staphylococcal enterotoxins A to E (*sea*, *seb*, *sec*, *sed*, and *see*) in multiplex PCR (Table-2). The conditions for PCR were described by Pyzik *et al*. [29].

**Table-2:** PCR conditions and primers used to detect toxicity genes in strains.

Primer	Oligonucleotide sequence (5’-3’)*	Gene	Size of amplified product (bp)	PCR conditions
ESA1 ESA2	ACGATCAATTTTTACAGC TGCATGTTTTCAGAGTTAATC	*sea*	144	Initial denaturation (5 min, 94°C); 30 cycles of amplification - denaturation 1 min *sec*, 94°C, annealing (1 min, 50°C), chain extension (1 min, 72°C); Final chain extension (2 min, 55°C); Final chain extension (5 min, 72°C); 4°C cooling
ESB1	GAATGATATTAATTCGCATC	*seb*	416	
ESB2	TCTTTGTCGTAAGATAAACTTC			
ESC1	GACATAAAAGCTAGGAATTT	*sec*	257	
ESC2	AAATCGGATTAACATTATCCA			
ESD1	TTACTAGTTTGGTAATATCTCCTT	*sed*	334	
ESD2	CCACCATAACAATTAATGC			
ESE1	ATAGATAAAGTTAAAACAAGCAA	*see*	170	
ESE2	TAACTTACCGTGGACCC			

* The primer concentration was 0.04 µmoL. PCR=Polymerase chain reaction

### Isolation and identification of parazites

Fresh fecal samples were obtained from the nests and from young falcons. The feces were mixed, placed in a Petri dish containing 2.5% (w/v) aqueous potassium dichromate (K_2_Cr_2_O_7_), and monitored daily. Oocysts were recovered by the flotation method with saturated saline solution (specific gravity=1.20) and examined microscopically. In addition, 2 g of fecal samples were used to determine the number of oocysts per g of feces (OPG ratio) by the McMaster method. The taxonomic classification of coccidian protozoa was based on morphological details of fully sporulated oocysts [30]. Measurements were made on 20 oocysts by differential interference contrast microscopy using Nikon NIS-Elements software.

### Detection of heavy metals

All heavy metals (Pb, Cd, arsenic [As], zinc [Zn], and Cu) in the feces and down feathers were analyzed by ICP-MS according to Lodenius and Solonen [31]. Determination of metals was preceded by ashing of all samples (dry heat incineration). For this purpose, 0.054-0.078 g of feathers from each individual, according to availability, and 2.427-2.924 g of feces were mineralized in an electric furnace at a final temperature of 450°C (dry heat incineration). The resulting ash was dissolved in 1M nitric acid, filtered (Whatman No. 42, Sigma Aldrich, USA), and made up to the 10 mL mark. To determine the validity of the method, the certified reference material DOLT-3 (dogfish liver, National Research Council of Canada, 11 Ottawa, Canada) was subjected to the same analytical procedure.

Heavy metals were determined with a Varian 820-MS ICP mass spectrometer, using the following parameters: Plasma flow 16 dm^3^/min, auxiliary flow 1.10 dm^3^/min, nebulizer flow 0.88 dm^3^/min, sampling depth 6.5 mm, RF power 1400 W, pump rate 6 rpm, and stabilization delay 40 s. Recoveries in the range of 90-110% were accepted as validation of the analytical procedure for the elements. The limits of detection obtained were 0.5, 0.25, 2.5, 0.25, and 4.0 mg/L^−1^ for Cd, Pb, As, Cu, and Zn, respectively.

### Statistical analysis

Statistical analysis of the concentrations of toxic elements was carried out by ANOVA in Statistica 10.0 software (Statistica, Poland). Because in a few cases, the concentrations of the metals were under the detection limit (Cd and As), Spearman rank correlation was calculated and tested. p≤0.05 was considered significant. The results were presented as means and standard deviations.

## Results

### Bacteria isolation and identification

Presumptive *E. coli* isolates (n=4) grown on MacConkey agar were obtained from three birds (one male and two female) from the nest in Lublin. No *E. coli* strains were isolated from the birds from Puławy. Four isolates (S1, S2a, Sa, and Sb) were correctly identified by MALDI-TOF-MS as *E. coli*. Log score values for all samples were >2, which indicates correct identification to species level (Table-3).

**Table-3:** Characterization of *Escherichia coli* strains isolated from peregrine falcons.

*Bird*	*Escherichia coli* isolate	Identification, log (score) value in MALDI TOF	Phylogenetic group	Phenotypic resistance	Resistance genes	Virulence genes
Male Lublin	Sa	2.133	A		
	Sb	2.232	B2	TE-R, CIP-I	*tetA*	*irp-2, pic*
Female 1 Lublin	S2a	2.021	A	TE-I	*tetA*	*astA*
Female 2 Lublin	S1	2.000	B2	nd	nd	*astA, irp-2, iss*

CIP=Ciprofloxacin, TE=Tetracycline, I=Intermediate, R=Resistant, nd=Not detected

**Figure-2:**
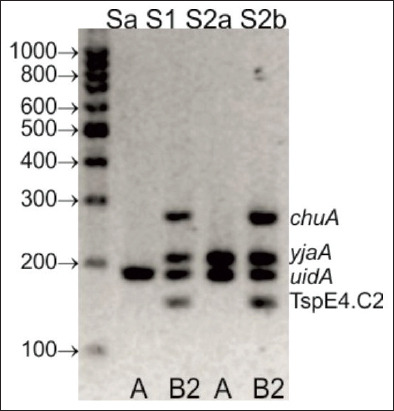
Polymerase chain reaction results for *Escherichia coli* isolates from peregrine falcons (*Falco peregrinus*).

Based on the electrophoretic profiles of multiplex PCR amplicons (*yja*, *TspE4.C2*, *chuA*, *svg*, and *uidA*), two *E. coli* isolates (Sa and S2a) were assigned to phylogenetic Group A and two isolates (S1 and S2b) to Group B2. Detailed PCR results are presented in Figure-2.

All four isolates were susceptible to the antibiotics tested, except for strain S2a, which showed intermediate susceptibility to tetracycline and isolate Sb, which was resistant to this antibiotic. The *tet*(A) gene was detected in both isolates (Table-3).

Of the 24 virulence genes considered in the study, only *astA* (encoding heat-stable enterotoxin 1 – EAST1), *irp-2* (encoding iron-repressible high-molecular-weight proteins), *iss* (encoding increased serum survival protein), and *pic* (encoding a serine protease precursor) were detected in three of the four *E. coli* isolates.

On the basis of morphological analysis, five *Enterococcus* spp. strains were isolated from all tested samples. Detailed MALDI TOF MS analysis revealed that the strains belonged to the species *Enterococcus hirae* (two strains) and *E. faecalis* (three strains). Both bacterial species were isolated from the samples obtained from falcons nesting in Lublin, whereas only *E. faecalis* strains were isolated from falcons nesting in Puławy. Log score values obtained in MALDI TOF for *Enterococcus* strains were >2.74 in the case of four strains and >1.7 for one strain, which indicates correct identification to species level (Table-4).

**Table-4:** Characterization of *Enterococcus* spp. strains isolated from peregrine falcons.

Bird sex and nesting site	*Enterococcus* species	DNA code	Identification log(score) value in MALDI TOF	Phenotypic resistance	Resistance genes	Virulence genes
Male, Lublin	*Enterococcus hirae* *Enterococcus faecalis*	L2a L2b P2	2.315 1.768 2.274	E15-I; MY15-R E15-I; MY15-R CIP5-R TE30-I TGC15-R S300-R MY15-R E15-I	nd nd *tet*-(L), *ant (4’)-Ia*	nd nd *asa1, gelE; efaAfs*
Female 1 Puławy	*Enterococcus faecalis*	P3a	2.330	CIP5-R; MY15-R TE30-I E15-I	*ant(4’)*-Ia; *Tn916/Tn1545*;	*gelE; efaAfs*
		P3b	2.274	CIP5-R; MY15-R TE30-I E15-I	*ant(4’)*-Ia; *Tn916/Tn1545*	*gelE; efaAfs*

TGC15=Tigecycline, E15=Erythromycin, S300=Streptomycin, MY15=Lincomycin, CIP5=Ciprofloxacin, TE30=Tetracycline, nd=Not detected

**Figure-3:**
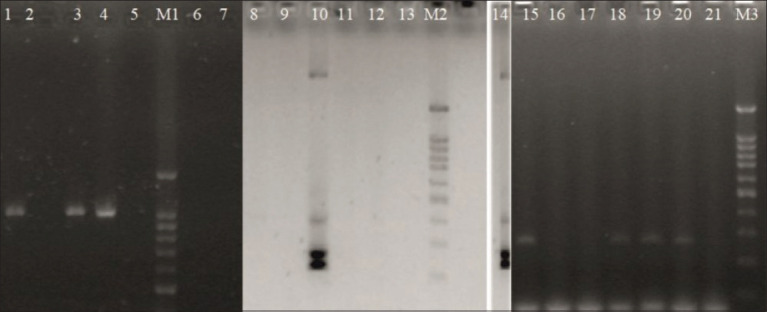
Resistance genes in *Enterococcus* spp. isolated from falcons. Lines: M1, M2, M3 - Marker Nova 100 bp DNA ladder; 1 – positive control *Enterococcus* *faecalis* UTI No. 1 for *Tn916/Tn1545* – 1028 bp; 2 - *E. faecalis* P2 (*Tn916/Tn1545*-); 3 – *E. faecalis* P3a (*Tn916/Tn1545*+); 4 – *E. faecalis* P3b (*Tn916/Tn1545*+); 5 – negative control for Tn*916*/Tn*1545 -* 1028 bp; 6 – *Enterococcus* *hirae* L2a (*Tn916/Tn1545*-); 7 – *E. hirae* L2b (*Tn916/Tn1545*-); 8 – *E. hirae* L2a (tetL-); 9 - *E. hirae* L2b (*tetL*-); 10 – *E. faecalis* P2 (*tetL*+); 11 – *E. faecalis* P3a (*tetL*-); 12 – *E. faecalis* P3b (*tetL*-); 13 – negative control *tetL*; 14 – positive control *E. hirae* WB No. 4 for *tetL*-229 bp; 15 – positive control *E. faecalis* WT No. 27 for *ant(4’)*-Ia -294 bp; 16 - *E. hirae* L2a (*ant(4’)*-Ia*-*); 17– *E. hirae* L2b (*ant(4’)*-Ia*-*); 18 – *E. faecalis* P2 (*ant(4’)*-Ia+); 19 - *E. faecalis* P3a (*ant(4’)*-Ia+); 20– *E. faecalis* P3b (*ant(4’)*-Ia+); 21– negative control.

The antibiotic resistance analysis confirmed resistance to lincomycin in all strains tested. All strains showed intermediate susceptibility to erythromycin. Strains identified as *E. faecalis* showed intermediate susceptibility to tetracycline and resistance to ciprofloxacin. No isolates were resistant to vancomycin, chloramphenicol, or any of the other chemotherapeutics (Table-4). Antibiotic resistance was linked to the *tet*(L) gene in one strain, to *ant(4’*)-Ia and *Tn916*/*Tn1545* in two strains, and to *efaAfs* in the three strains of *E. faecalis*. No drug resistance genes were shown in *E. hirae* strains (Table-4). A detailed analysis of the presence of resistance genes is presented in Figure-3.

**Figure-4:**
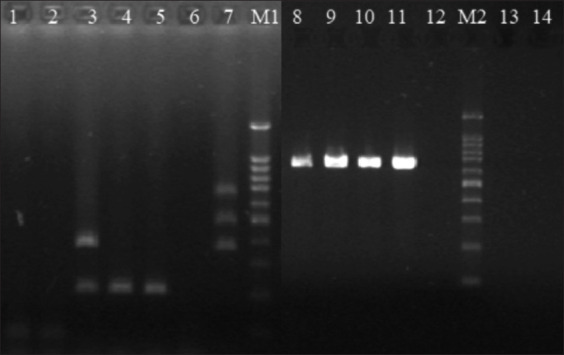
Virulence factors in *Enterococcus* spp. isolates from peregrine falcons. Lines: M1, M2 - Marker Nova 100 bp DNA ladder; 1 – *Enterococcus hirae* L2a; 2 - *E. hirae* L2b; 3 – *Enterococcus* *faecalis* P2 (*asa1* +, *gelE*+); 4 – *E. faecalis* P3a (*gelE*+); 5 – *E. faecalis* P3b (gelE+); 6 – negative control; 7 – positive control *E. faecalis* WT3 for genes: *cylA* - 688 bp; *esp* - 510bp; *asa1* – 375 bp; 8 – *E. faecalis* P2 (*efaAfs*+); 9 – *E. faecalis* P3a (*efaAfs*+); 10 – *E. faecalis* P3b (*efaAfs*+); 11 – positive control *E. faecalis* ATCC29212 (*efaAfs*-); 12 – negative control; 14M –13 – *E. hirae* L2a (*efaAfs*-); 14 - *E. hirae* L2b *(efaAfs*-).

The PCR analysis of virulence factors in *Enterococcus* spp. confirmed the simultaneous occurrence of the gelatinase gene (*gelE*) and cell wall adhesin gene (*efaAfs*) in the three *E. faecalis* strains (P2, P3a, and P3b) isolated from chicks nesting in Lublin and Puławy, as well as the aggregation protein gene (*asa1*), which was present only in *E. faecalis* strain P2 (Figure-4). The presence of virulence factors was not confirmed in the *E. hirae* strains.

*Staphylococcus* species were isolated only from nestlings in Puławy, based on morphological analysis confirmed by MALDI TOF MS. In total, three strains belonging to the species *Staphylococcus schleiferi* and one strain of *S. aureus* obtained from the male were isolated. Log score values in MALDI TOF for *Staphylococcus* spp. strains were >2.0 in the case of three strains and >1.95 for one strain, which indicates correct identification to species level (Table-4).

The strains did not show resistance to the chemotherapeutics used. Intermediate susceptibility to clindamycin was observed in only one *S. schleiferi* strain isolated from female 1. Both strains isolated from the male showed intermediate susceptibility to erythromycin (Table-5). The analysis of resistance genes also did not confirm the presence of any of them.

**Table-5:** Characterization of *Staphylococcus* spp. strains isolated from peregrine chicks.

Bird sex and nesting site	Staphylococcus species	DNA code	Identification log(score) value in MALDI TOF	Phenotypic resistance	Resistance genes
Female 1 Puławy	*Staphylococcus schleiferi*	S1E	2.029	DA2- I	nd
Female 2 Puławy	*Staphylococcus schleiferi*	2E2	2.037	nd	nd
Female 3 Puławy	nd	-	-	-	-
Male 1 Puławy	*Staphylococcus schleiferi*	3E3a	1.959	E15-I	nd
	*Staphylococcus aureus*	3E3b	2.042	E15-I	nd

E15-I=Erythromycin intermediate resistance, DA2-I=Clindamycin 2 intermediate resistance, nd=Not detected

**Figure-5:**
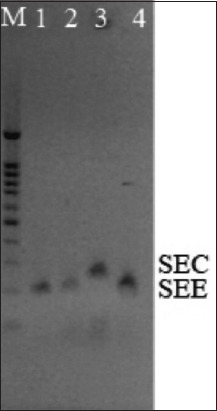
Results of the detection of enterotoxicity genes in *Staphylococcus* spp. isolates from peregrine falcons (*Falco peregrinus*). M - mol. weight standard 100-1000 bp and 1500 bp (Nova 100 bp DNA Ladder, 250); Line 1 - *Staphylococcus schleiferi* S1E, Line 2 - *S. schleiferi* 2E2; Line 3 - *S. schleiferi* 3E3a; Line 4 - *Staphylococcus aureus* 3E3b.

The results of multiplex PCR showed the presence of SEE genes (170 bp) responsible for the production of enterotoxin E in two strains of *S. schleiferi* subsp. *schleiferi* (S1E and 2E2), isolated from females 1 and 2, respectively, and in the strain of *S. aureus* subsp. *aureus* (3E3b) from the male. In addition, the presence of the SEC (257 bp) gene responsible for the production of enterotoxin C was confirmed in the *S. schleiferi* strain isolated from the male (Figure-5).

### Parasitic identification

**Figure-6:**
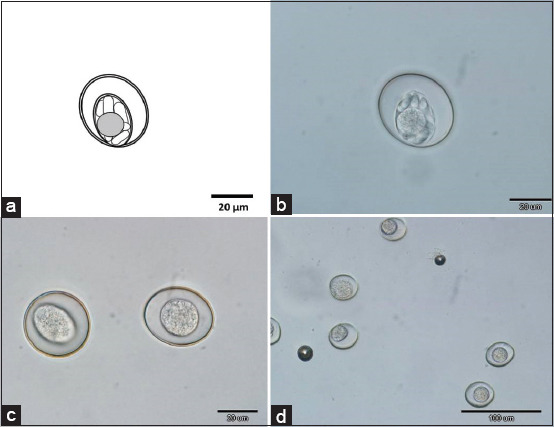
*Cyclospora falconis* from young peregrine falcons. Line drawing of mature oocyst, showing details: (a) Subspherical sporocyst with banana-shaped sporozoites and spherical residuum. (b) The same living oocyst as in the line drawing in differential interference contrast light microscopy, (c and d) unsporulated oocysts isolated from fresh fecal samples.

Only one species of coccidia was found in the material; it was identified as *Caryospora falconis*. The intensity of infection was 1800 OPG. Oocysts (n=20) were spherical to subspherical, ranging in size from 31.5 μm to 36.5 μm (average 32.5×34.0 μm), with smooth bilayered walls without micropyles. Single sporocysts containing eight sporozoites were spherical (average diameter 21 μm), with no Stieda body and with a sporocyst residuum present as a diffuse spherical mass (Figure-6).

### Heavy metals analysis

Toxicological analysis of feather and fecal samples obtained from the falcons revealed high concentrations of Cd, Cu, Pb, Zn, and As in all samples. The highest concentrations in both fecal and feather samples were observed for Cu (11.33 μg/g and 15.42±0.83/g of sample, respectively) and Zn (42.05 and 22.04 μg/g of sample). The average values of the elements exceeded the concentrations detected and recognized as toxic in other studies even ten-fold. Detailed data are presented in Table-6 [32-35].

**Table-6:** Average concentrations of Cd, Cu, Pb, As, and Zn in samples obtained from peregrine chicks.

Element	Concentration µg/g		*P*≤0.05	Range in studies by other authors µg/g	References

	Feces	Feathers			
Cd	0.319±0.036	<0.001	0.0001	0.63-0.76 >0.08	Mansouri and Majnoni, [32]; Ek *et al*. [34]
Pb	2.46±0.1	1.08±0.05	0.0002	1.3-2.43 >0.09	Gruz et al. [33] Ek *et al*. [34]
Cu	15.42±0.83	11.33±0.4	0.0002	5.28-8.33 >8	Ek *et al*. [34]
Zn	42.05±6.42	22.04±1.57	0.00015	24.12-25.77 >45	Ek *et al*. [34]
As	0.04±0.003	<0.005	0.07	0.161>200	Burger, [35]

Cd=Cadmium, Pb=Lead, As=Arsenic, Zn=Zinc, Cu=Copper

## Discussion

Free-living birds nesting in urban ecosystems may be particularly exposed to contact with common zoonotic pathogens posing a threat to animals and humans. In addition, they are particularly vulnerable to poisoning with heavy metals arising during manufacturing processes. Numerous studies [32,33] confirm the large role of pollutants associated with highly urbanized and industrialized regions in the accumulation of heavy metals and other toxic compounds in birds. The presence of bacterial agents in free-living birds as a potential reservoir of pathogens has also recently become a subject of research, due in part to the potential threat posed to domestic animals and humans [19].

The present study found pathogenic *E. coli*, *Enterococcus* spp. and *Staphylococcus* spp. bacteria and *C. falconis* parasites in peregrine falcons nesting in urban environments. It should be emphasized that single virulence genes were found among the bacterial isolates: (*irp-2*, *pic*, *astA*, and *irp-2*) in *E. coli* belonging to phylogenetic groups A and B2; enterotoxicity genes (SEC and SEE) in *Staphylococcus* spp.; and three genes coding for virulence factors (*asa1*, *gelE*, and *efaAfs*) in *Enterococcus faecalis*. Similar results for the occurrence of bacterial strains in wild birds were observed in a study by Chung *et al*. [2], in which the most frequently identified bacteria were *E. coli* and *Campylobacter jejuni*. In another study [36], *E. coli* isolates from wild birds were also mainly assigned to phylogenetic Groups A and B2. According to these authors, the prevalence of a given species may depend on the geographic zone, the species and order of bird, the migratory status of the bird species, its habitat type, and the species, family, and phylum of bacteria associated with the bird.

A positive finding of our research was the low resistance of the bacterial isolates to the antibiotics tested. In the case of *E. coli* strains, resistance to tetracycline was found in only one isolate, which was also confirmed by the presence of the tetracycline resistance gene *tet*(A) in two of the four isolates. The presence of tetracycline resistance genes in *E. coli* strains isolated from wild birds has also been demonstrated by other authors [36].

Bacteria of the genus *Staphylococcus* did not show resistance to the antibiotics, which was linked to the lack of resistance genes confirmed by PCR. In the case of these two nests, the peregrine falcons were shown not to be exposed to drug-resistant *Staphylococcus* bacteria posing a potential threat to birds and the environment, which is a highly favorable finding. The results differ from those reported by Chung *et al*. [2], who detected various antibiotic resistance genes in *Staphylococcus* spp. isolated from wild birds. In our study, however, samples were collected from only two nests.

By far, the most numerous source of resistance was *Enterococcus* spp. strains, among which ­resistance was demonstrated against six of the 17 antibiotics (tetracycline, tigecycline, erythromycin, streptomycin, lincomycin, and ciprofloxacin). This was linked to the presence of as many as five resistance genes among the strains isolated from chicks.

High resistance to tetracycline (about 71%) in bacteria isolated from other species of wild birds, including as migratory and non-migratory birds, especially among strains of *Staphylococcus* spp., *E. coli*, and *Enterococcus* spp., was observed in an extensive analysis presented by Chung *et al*. [2]. Other studies [1,37], conducted in wild geese, cormorants, and birds of prey, have also confirmed resistance to beta-lactam antibiotics in *E. coli* strains, which was confirmed by the presence of resistance genes, including tet(A)/tet(B), aadA, sul1/sul2/sul3, and qnr. In our study, the presence of a resistance gene in *E. coli* strains was demonstrated only for tetracycline, which may be due in part to the very small number of strains obtained from the six chicks (n=4). However, the resistance to tetracycline shown in our research is consistent with the results of the works cited above and confirms the occurrence of resistant strains as well as tetracycline resistance genes among bacteria isolated from wild birds.

The largest group of resistant microorganisms isolated from samples obtained from peregrine falcon chicks was strains of *Enterococcus* spp. The resistance to tetracycline, erythromycin, lincomycin, ciprofloxacin, tigecycline, and streptomycin demonstrated in the isolates represents a serious threat not only to domestic and livestock animals but also to people as well. Our previous work [15] demonstrated a high percentage of strains resistant to lincomycin (100%), tetracycline (48%), and erythromycin (44%) among isolates from other species of free-living birds. These results also confirm reports by Marrow *et al*. [38], who showed a very high prevalence percentage (95%) of *E. faecalis* strains in wild birds and a high percentage of resistance to second-generation cephalosporins and erythromycin. Similar results are reported in a study by Radhouani *et al*. [36], in which 87.1% of *Enterococcus* spp. isolates were resistant to tetracycline and 80.6% to erythromycin. In addition, about 30% of the strains were resistant to ciprofloxacin.

The presence of resistance genes in the *E. faecalis* strains isolated from peregrine falcons, which determine resistance to tetracycline, lincomycin, erythromycin, ciprofloxacin, and tigecycline, also confirms the results of previous research [39,40], in which samples from birds of prey as well as from passerines and waterfowl were tested. In that study, most of the resistance genes were found in the species *E. faecalis*, which is also consistent with results reported by Radhouani *et al*. [36]. The presence of such a large population of genes determining resistance to the above-mentioned antibiotics among *Enterococcus* strains suggests that free-living predators are potential indicators of these genes.

Parasitological analysis of the material obtained from the peregrine falcon chicks confirmed the presence of the coccidian parasite *C. falconis* in numbers indicating moderate intensity of infection (1800 OPG). *Caryospora* is the third largest genus in the family *Eimeriidae*, and its members infect primarily predatory birds and reptiles [41]. At least 25 species of *Caryospora* have been identified from wild birds [42]. To date, 15 species have been identified in raptor birds worldwide, while nine species have been recorded only in falconids from Asia, Europe, and North America. Members of the genus *Caryospora* are the most important cause of gastrointestinal disorders in falcons [43,44], especially in young birds from breeding facilities. The host specificity of *C. falconis* is difficult to determine; it is unclear whether it is a species-specific parasite. Thus, far *C. falconis* has been found in *F. peregrinus*, *Falco subbuteo*, and *Falco tinnunculus* in the European population.

A high level of immunity plays an important role in resistance to bacterial and parasitic infections in wild birds. Many factors, including heavy metals, primarily As, Pb and Cd, Zn, and Cu, can significantly limit natural immune mechanisms in birds, increasing their susceptibility to infection. Data presented by other authors [32,32,45] indicate that the concentration of heavy metals is dependent on the nesting and migration sites of birds, as well as their individual and species characteristics, including species resistance, age, sex, and health status. In many cases, close ­proximity of birds to highly urbanized areas is an important factor in increasing the accumulation of these elements in the internal organs (liver, kidneys, and lungs) and skin appendages (feathers and talons), thereby weakening the birds [46].

Our study found high concentrations of Pb, Cu, and Zn in the feces (2.26, 15.42, and 42.05 μg/g, respectively) and feathers (1.08, 11.33, and 22.04 μg/g) of peregrine chicks. These concentrations are higher than those reported in other studies [32,33], which were considered toxic for predatory birds, including peregrines. In the case of Cd and As, the concentrations were lower than those found in other studies [32,34,45].

Various ranges of heavy metal concentrations have been found in the feathers of free-living birds; in penguins, for example, Cd levels ranged from < 0.01 μg/g in the young to 1.9 μg/g in adult birds living in the vicinity of Iceland and King George Island [47]. Licata *et al*. [45] showed that a high percentage of deaths in buzzards are due to chronic heavy metal poisoning, for example, with Pb or Cd. Based on the accumulation properties of the heavy metals, the authors established toxic values from 0.10 μg/g for Cd and from 1.60 μg/g for Pb. The concentrations of 0.319 μg/g for Cd and 2.46 μg/g for Pb in our study clearly represent a level of toxicity that could affect the health status and welfare of these birds. The high Zn level, particularly in the feathers of the chicks (42.05 μg/g), seems to be a cause for concern, as it significantly exceeded the value of 24.1 μg/g obtained by other authors [32,47]. Moreover, due to the cumulative properties of Zn, this element should be taken into account in the assessment of the risk for birds, especially chicks living in urban areas.

## Conclusion

To sum up, the results of the study indicate that free-living birds are biological indicators of environmental pollution, antimicrobial resistance, and resistance genes. In addition, high concentrations of heavy metals and other toxic elements may have affected their health and welfare during the rearing period.

## Authors’ Contributions

EP: Conceptualization; data curation, material collection and initial isolation of bacteria. MD: Methodology and characterization of *E. coli* bacteria, writing of *E. coli* methodology and figure preparation. DS: Methodology and characterization of *Enterococcus* spp., writing of *Enterococcus* spp. methodology. AM: Methodology and characterization of *Staphylococcus* spp., JLVP: Participation in toxicology analysis. AC: Participation in toxicology analysis, with resources and writing of this part of the manuscript. KS: Parasitology testing with writing and visualization of this part of the manuscript. RU: Project administration, resources, writing – review and editing, software; statistical analysis, preparation of tables and figures. All authors read and approved the final manuscript.
